# Applying the Dam to Difficult Cervical Cavities*Extracted from “Around the Table,” in the October Dental Items of Interest.

**Published:** 1917-11

**Authors:** R. Ottolengui


### APPLYING THE DAM TO DIFFICULT CERVICAL CAVITIES *

BY R. OTTOLENGUI, D.D.S.

We can not use a clamp without injury to soft tissues, where the cavity margin is below the free margin of the gum, unless you force the gum away. As a matter of fact, the primary object of forcing the tissues away from the cavity margin is that the margin itself may be prepared and the clamp adjusted without injury to the gum.

Dealing with specific cases, the cavities must be divided into two classes; those so situated that the clamp must be placed upon the tooth in which the cavity is found; and, second, those in which the clamp may be adjusted about some other tooth. In this latter class of course the possible injury by the clamp has no relation to the cavity from which tissue has been forced away, because the clamp is on some adjacent or even distant tooth. But you may say that it is just as bad to injure the cingulum about a sound tooth as about one that has a cavity. There are many teeth about which a clamp can be adjusted without injury by the usual methods, but it can be done as follows:

Suppose the tooth to be filled is a first upper bicuspid. Punch four holes and slip these over the lateral incisor, the cuspid and both bicuspids. With a smooth-edged burnisher evert the edges of the dam to fold beneath the free margin of the gum. Now draw the rubber dam tightly over the molar tooth, just as though there were a hole in the rubber, only there is no hole. When this is stretched so as to fully disclose the molar tooth under the rubber, adjust a molar clamp, one of the proper form, over the rubber dam which is over the molar. The dam is nicely held in

* Extracted from "Around the Table," in the October Dental Items of Interest.

place, and the clamp neither injures the gum nor causes pain.

The difficult cases are those where we must apply the clamp to the tooth in which the cavity exists. And this clamp must be used so that the patient will not call it the "dam clamp." In other words, the saliva must be dammed, but not the clamp. The trouble with most cervical clamps is that they have two sharp points, usually the terminals of a semilunar edge, which none but a loon would expect would fit more than one or two teeth in a hundred. Usual result is that one sharp point on the tooth and the other is hurting and injuring the soft tissue.

Reshape this clamp! Grind the two points into a single point. Make this sharp and slightly curved gumwards. Suppose now that we have used temporary stopping to force the gum away from the gingival margin of a labial cavity in a lateral incisor. Removing the temporary stopping, we note that the cavity margin is now nicely disclosed. If not, a second treatment with the temporary stopping would be expedient. Holes are properly placed in the dam, so that when in position the interproximal gum tissue would be covered by the dam. This necessitates placing one hole slightly out of alignment. The dam is drawn over, say four teeth, and stretched so as to disclose the gum that has been pressed out of the cavity. The tender gum then being in plain view, the single-toothed clamp is carefully placed so that the sharp point of the clamp rests upon the tooth neck just above the cavity margin, but does not impinge upon the soft tissue. Warm air is now used and the parts thoroughly dried. Then, if desirable, with a stout instrument you may press gently but firmly against the clamp, forcing it higher up, disclosing more of the tooth neck above the cavity margin and compressing but not injuring the soft tissue. This can and should be done painlessly, without shedding a drop of blood.

In cases where the operation will consume but a short time, and no cervical clamp is at hand which will properly fit at both the labial and palatal surfaces of the tooth, a not uncommon circumstance, the dam can be kept above the cavity margin without injury to tooth or gum by gently forcing the dam to place with a steel needle or pin, and driving the point of the needle into the dentin above the cavity margin.

By another method we may achieve the same result. A silk, well waxed, may often be tied above the cavity margin. In such case the end of the silk should be left six to eight inches long, and may be passed up over the forehead, a napkin protecting the face, and a heavy weight attached to it will hold the dam in place nicely, while giving free use of the hands.

For a cavity in the buccal surface of a second lower molar; very sensitive and mouth very wet. Packed gum out of cavity and well away from cavity margin, by two treatments, using temporary stopping, tied in with silk; then softened with warm burnishers and pressed home.

When the gingival margin was well disclosed, a copper band, just a trifle too small in circumference, was forced over the molar and manipulated with band-forcing instruments (such as are used by orthodontists), so that the band was actually stretched to place. By this means it passed beneath but did not impinge upon the gum margin. In the buccal region, of course, the first application of the band covered and consequently hid the buccal cavity from view. This part of the band was cut away sufficiently to disclose the upper half of the cavity. The band was then forced back to place. The rubber was put on and the clamp adjusted without injury or pain, after which the upper edge of the copper band, which still hugged the region of the cavity somewhat, was forced outwardly, the soft metal yielding nicely, and the cavity was then readily prepared and filled. And I believe I may say prepared and filled with that thoroughness that never could have been accomplished without the dam.

With the rubber dam in place I easily used chlorid of ethyl and the preparation of the cavity was absolutely painless. Of course there was some little shock and pain when using the chlorid of ethyl, but that is momentary and transitory.

Approximal cavities extending below the gum margin occur either in teeth having living pulps or in teeth in which canal treatment is requisite. In the latter case the rubber dam is absolutely a prerequisite to anything even approximating an aseptic operation. The recourse then must be the use of the copper band. The gum is generally inflamed, or otherwise diseased when we start treatment. We need only to inaugurate a technic which will not increase the disturbance, but rather offer opportunity for improvement.

In fitting the copper band, select one a bit too small rather than one a bit too large. Festoon the band at the interproximal aspect so that it may be forced beyond the interproximal cavity margin, without injury to the gum tissue elsewhere about the tooth neck. The extreme edges of the band should be inverted, bent toothwards, so that it shall present a rounded edge to the soft tissues. A properly formed and fitted band cemented to place and left on until all canal treatment and filling shall have been completed will usually give the gum ample opportunity to heal, and when removed will not only disclose the cavity margin, but permit preparation of this margin without injury to the gum.

It will often occur, however, that the rescession of the gum interproximally will be so deep that if the dam is used the septum of rubber lying between the teeth can not be made to dip downwards to include the cavity margin. This sort of cavity, as well as those in which the pulps are found alive, and which are to be filled without resort to the copper band, may both be managed in like manner.

Such cavities usually being large, are not nowadays filled with anything but gold inlays or amalgam. Hence absolute dryness for an extended period is not necessary, as it would be for a gold foil filling. The technic, therefore, is quite simple. A single hole in the dam, made with the largest punch, is stretched over two teeth, the one with the cavity and the one immediately adjacent thereto. Thus the cavity margin is in plain view, the cavity is kept quite sufficiently dry, the lips and tongue are kept out of the way, and the operation proceeds without injury to the tissues.

